# Synthesis and Disinfection Effect of the Pyridine-4-aldoxime Based Salts

**DOI:** 10.3390/molecules20033681

**Published:** 2015-02-24

**Authors:** Jan Marek, David Malinak, Rafael Dolezal, Ondrej Soukup, Marketa Pasdiorova, Martin Dolezal, Kamil Kuca

**Affiliations:** 1 Department of Medicinal Chemistry and Drug Analysis, Faculty of Pharmacy, Charles University in Prague, Heyrovskeho 1203, Hradec Kralove 500 05, Czech Republic; E-Mails: marekjanmgr@seznam.cz (J.M.); martin.dolezal@faf.cuni.cz (M.D.); 2 Department of Epidemiology, Faculty of Military Health Sciences, University of Defence, Trebesska 1575, Hradec Kralove 500 01, Czech Republic; E-Mail: MarketaPasdiorova@seznam.cz; 3 Biomedical Research Centre, University Hospital Hradec Kralove, Sokolska 581, Hradec Kralove 500 05, Czech Republic; E-Mails: david.malinak@gmail.com (D.M.); rafael.dolezal@fnhk.cz (R.D.); soukup.ondrej07@gmail.com (O.S.); 4 Department of Cybernetics and Biomedical Engineering, Faculty of Electrical Engineering and Computer Science, VSB-Technical University of Ostrava, 17. Listopadu 15, Ostrava-Poruba 708 33, Czech Republic; 5 Department of Toxicology and Military Pharmacy, Faculty of Military Health Sciences, University of Defence, Trebesska 1575, Hradec Kralove 500 01, Czech Republic

**Keywords:** pyridinium-4-aldoxime salts, synthesis, analysis, antimicrobial activity, surfactants, cytotoxicity

## Abstract

A set of new quaternary ammonium compounds based on pyridine-4-aldoxime was synthesized, characterized with analytical data (NMR, EA, HPLC, MS) and tested for *in vitro* antimicrobial activity (antibacterial, antifungal) and cytotoxicity. Quaternary pyridinium-4-aldoxime salts with length of alkyl side chain from C8 to C20 and belonging to the group of cationic surfactants were investigated in this work. An HPLC experimental protocol for characterization of mixtures of all homologues has been found. Antimicrobial evaluation found that yeast-type fungi were most sensitive towards C_14_ and C_16_ analogues, whereas the C_16_ analogue was completely ineffective against filamentous fungi. Antibacterial assessment showed versatility of C_14_ and relatively high efficacy of C_16_ against G+ strains and C_14_ against G− strains. Notably, none of the studied compounds exceeded the efficacy and versatility of the benzalkonium C_12_ analogue, and benzalkonium analogues also exhibited lower cytotoxicity in the cell viability assay.

## 1. Introduction

The 20th century can appropriately be called the age of organic chemistry. It brought many new organic structures, both natural and synthetic. Here the discussed surfactants are undoubtedly a huge group of organic compounds for which a technical use has been found. These compounds have certainly undergone considerable development since their discovery in 1930, and countless applications have been reported for these important molecules (e.g., detergents, disinfectants, decontaminants, *etc.*) [1,2,3].

Cationic surfactants consist of a hydrophilic part, such as a quaternary nitrogen moiety, able to interact with polar chemical milieu, and a hydrophobic part (e.g., a long alkyl chain), which can, on the other hand, penetrate into non-polar molecular agglomerates.

Many types of cationic surfactant based on quaternary nitrogen have been described [4,5,6]. One important property described for quaternary ammonium salts is inhibition of the growth of bacteria and fungi. The most commonly used are derivatives of pyridinium, cetrimonium, benzalkonium and benzoxonium salts [7,8,9,10]. Some pyridinium salts are already in use as disinfectants in many preparations (as a component of eye drops, solutions, disinfection foams, *etc.*). Currently, there have been many articles describing cetylpyridinium salt as a protective agent against food-borne disease [6]. Recently, polymeric quaternary ammonium salts have been used in the development of bactericidal surfaces [11,12]. Another antimicrobial application of cetylpyridinium is its addition to chewing gums or dental formulations as an antiplaque agent [13,14]. In order to intensify the development of cetylpyridinium analogues, the 2D and 3D QSAR method was established [6]. It was found that numerous properties such as substituents on the pyridine moiety, alkyl side-chain length, hydrophobicity, pKa and absorbability on to the cell are critically important for antimicrobial activity [15,16].

**Figure 1:**
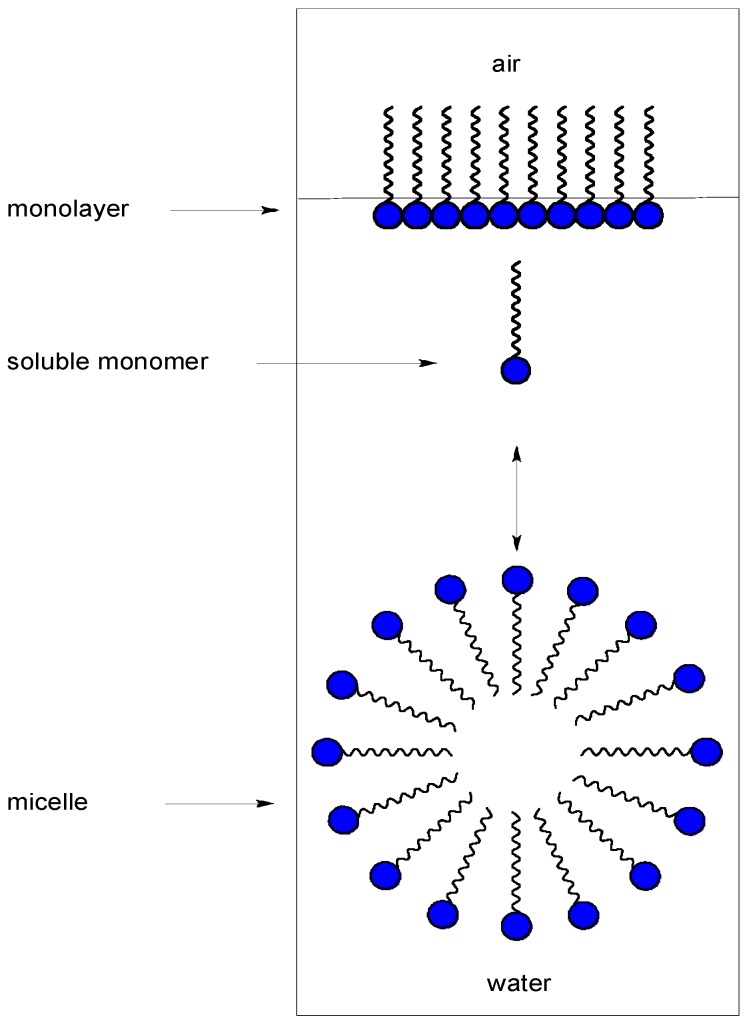
Micelles.

Quaternary ammonium salts (QAS) are also frequently used in prevention of nosocomial infections that are predominantly acquired within healthcare facilities, and conditioned mainly by the impaired immune status of patients, absence of a strict glove-change regime, and the use of improperly or inadequately sterilized medical instruments [17]. An important property of such compounds is their ability to form micelles (Figure 1). These formations are created mainly in aqueous solution, when the critical micelle concentration is exceeded. Many cationic surfactants can also be used as micellar catalysts [18], able to accelerate chemical decomposition [19,20], and such compounds can find use as decontaminants for military purposes. Thus these properties are important in decontamination procedures for measuring the kinetics of cleavage of some model compounds such as organophosphates and others [21,22,23,24,25,26]. Many other properties of cationic surfactants were discovered. Enthalpies of dilution, density effects of counterions in micellar solution and osmotic coefficients have been measured as a function of concentration, and the results expressed in terms of partial molar quantities [27,28,29].

Our group has been devoted to the synthesis and evaluation of derivatives of heterocyclic nitrogen compounds [30,31,32,33] and tensides [34,35,36]. Preparation of the various pyridinium-4-aldoxime salts has already been described before by our group, where the compounds were evaluated as micellar catalysts [24,25]. However, there has been no description of the synthesis of the whole series of such salts differing in the alkyl chain (C_8_ to C_20_), and their antimicrobial properties have not been measured. The primary aim of this work was to investigate this group of QAS as potential affordable disinfectants. For this reason a universal method was developed for preparation of monoquaternary pyridinium-4-aldoxime salts with alkyl-chain substituents, and the prepared compounds were characterized using analytical data (NMR, EA, HPLC, MS) and tested for *in vitro* antimicrobial activity and cytotoxicity.

## 2. Results and Discussion

### 2.1. Synthesis and Analysis

**Scheme 1:**
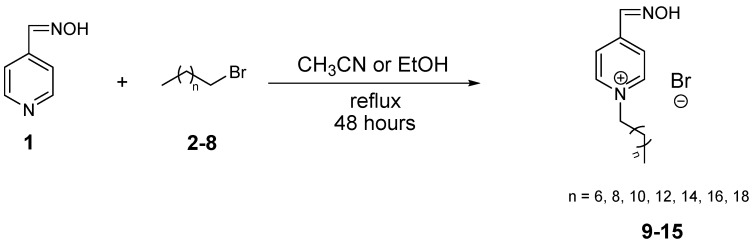
Preparation of pyridinium-4-aldoxime salts.

The results achieved within our study are shown in Table 1. This includes yields, melting points, retention times obtained on HPLC and calculated log*P* (Clog*P*). It is evident that the preparation of pyridinium-4-aldoxime salts with side chain C_8_–C_20_ is a quite elementary one step reaction. On the other hand, repeated crystallization was necessary in order to achieve the required purity. Two different conditions for synthesis were used. The reactions were realized in two different solutions, ethanol (EtOH) or acetonitrile (CH_3_CN) (Scheme 1), and the prepared compounds **9**–**15** were recrystallized from ethyl acetate or acetone. There was no significant variation in the yields from both solvents. Yields were highest for compounds **11**–**13**. A comparison can be made of the influence of the non-polar part of molecule on the yields during the quaternization of *N,N*-dimethyl-*N*-benzylamine, pyridine or derivatives of quinoline [31,33,37,38]. As expected, the value of Clog*P* increases with the length of the non-polar chain.

**Table 1:** Yields, melting points and retention times of prepared pyridinium-4-aldoxime salts.

Comp.	R	Yields (%) EtOH	Yields (%) CH_3_CN	m.p. (°C)	HPLC Rt (min)	Clog*P*
**9**	C_8_	15	X	92–93	4.15	0.04
**10**	C_10_	27	41	130–132	4.67	1.10
**11**	C_12_	70	55	136–137	5.22	2.15
**12**	C_14_	72	75	144–146	5.82	3.21
**13**	C_16_	75	87	144–145	6.49	4.27
**14**	C_18_	47	76	128–130	7.23	5.33
**15**	C_20_	45	X	126–128	8.06	6.39

Note: X—Not prepared in CH_3_CN.

**Figure 2:**
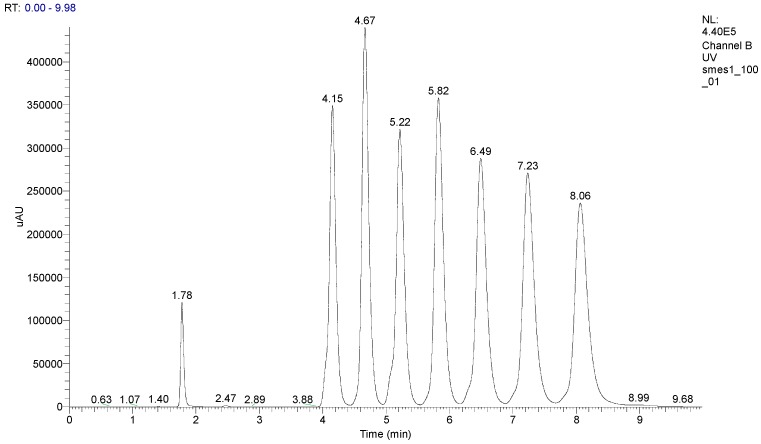
HPLC chromatogram of pyridinium-4-aldoxime salts mixture.

Additionally, HPLC analysis was carried out for individual compounds. A newly developed HPLC method was able to distinguish all prepared quaternary pyridinium-4-aldoxime salts (Figure 2). The shortest retention time was found for the C_8_ pyridinium-4-aldoxime salt. This novel HPLC method could be easily used for characterization of mixtures of all homologues.

### 2.2. Antimicrobial Activity

A set of eight fungus strains was used to evaluate antifungal activity. The minimum inhibitory concentrations (MICs) and minimum fungicidal (MFCs) concentrations for four yeasts and four filamentous fungi are summarized in Table 2. The whole set of pyridinium-4-aldoxime salts was tested for biological activity. The results for compounds **9**–**15** as well as for the reference benzalkonium B_12_, B_14_ and B_16_ compounds are shown in Table 2.

A broad spectrum of activity is evident for compounds **11**–**14**. The CK strain is sensitive for the whole set of pyridinium-4-aldoxime salts except for compound **15** (C_20_ alkyl chain). The yeast-type strains (CA, CT, CK, CG) are most sensitive to compounds **12**–**14**. The effectiveness is comparable with the benzalkonium salts. However, there is no clear correlation with the alkyl chain length.

The disruptive effect of QAS on the microorganism is probably based on the adsorption of this amphiphile molecule on the outer cellular membrane. The positively charged “heads” of the cationic molecules interact with the negatively charged cell membrane, disrupting it thanks to electrostatic and hydrophobic interactions. These interactions effectively out-compete the divalent cations, which normally stabilize surface structures by linking adjacent negatively-charged components [39]. Once close contact is accomplished by the hydrophilic region, the hydrophobic region proceeds to penetrate the hydrophobic bilayer to cause cell leakage and lysis [40]. This cascade leads to the release of K^+^ and cytoplasmic components, and finally to death of the cell. The antibacterial activity thus depends on the hydrophilic-hydrophobic balance of the cationic surfactants. Increase in the alkyl chain length (from C_12_ to C_16_) increases the hydrophobic character, which may be too high to facilitate transport through the bacterial cell membrane [41].

The filamentous fungi strains have significant sensitivity only to compounds **12** and **13** (alkyl length C_14_ and C_16_). The values of MICs and MFCs are higher compared to benzalkonium reference salts. The other compounds have no or only minor efficacy against filamentous fungi.

The *in vitro* antibacterial activity of compounds **9**–**15** was also assayed for the eight strains of bacteria. Gram positive (G+) and gram-negative (G−) bacteria groups are represented to cover the entire spectrum. Listed in Table 3 are the obtained minimum inhibitory concentrations (MICs) after 24 h and 48 h of incubation, and the minimum bactericidal concentrations (MBCs) after 48 h of incubation. Again, commercially used benzalkonium salts with alkyl side-chain length C_12_, C_14_ and C_16_ are included for comparison of antibacterial activity (B_12_, B_14_, B_16_).

In general, the relationship of structure and antibacterial activity shows the most efficient compounds to be **11**–**13** (alkyl chain C_12_, C_14_, C_16_), highlighting the versatility of **12** and the high efficacy of **13** against G+ strains. The other compounds with a shorter or longer lipophilic chain did not show any distinctive effect. Only the relatively high potency of **11**, **14** and **15** against SE can be noted. It was found that the Gram-positive bacteria (first four strains) are most sensitive to compounds **12** and **13** (alkyl chain C_14_ and C_16_). In comparison with the benzalkonium salts, the effectiveness of compound **13** seems to be better (MBC) against SA. The sensitivity of the other strains of G+ bacteria is comparable or lower.

**Table 2:** Minimum inhibitory/fungicidal concentrations of the prepared compounds (**9**–**15**) and selected derivatives of *N*-benzalkonium (B_12_–B_16_). The *in vitro* activities were determined on a panel of eight fungal strains.

Microorganisms	MIC (μmol/L); 24 h/48 h Incubation									
	MFC (μmol/L); 48 h Incubation									
	9	10	11	12	13	14	15	B_12_^a^	B_14_^a^	B_16_^a^
*Candida albicans* ATCC 44859 (CA)	>500/>500	125/250	15.62/15.62	1.95/1.95	3.9/3.9	7.81/7.81	>500/>500	0.49/0.49	7.81/7.81	3.91/7.81
	>500	250	15.62	1.95	3.9	15.62	>500	0.98	125	7.81
*Candida tropicalis* 156 (CT)	125/125	31.25/31.25	7.81/7.81	3.9/3.9	3.9/3.9	7.81/7.81	>500/>500	0.49/0.49	3.91/3.91	3.91/7.81
	250	125	31.25	15.62	3.9	15.62	>500	0.98	125	7.81
*Candida krusei* E28 (CK)	31.25/62.5	3.9/3.9	0.98/1.95	1.95/1.95	3.9/ 3.9	3.9/3.9	>500/>500	0.49/0.49	3.91/3.91	1.95/1.95
	125	15.62	3.9	15.62	3.9	15.62	>500	0.49	125	1.95
*Candida glabrata* 20/I (CG)	125/125	31.25/31.25	3.9/3.9	1.95/3.9	3.9/3.9	3.9/3.9	>500/>500	0.49/0.49	7.81/7.81	1.95/3.91
	250	62.5	3.9	31.25	3.9	7.81	>500	0.49	125	62.5
*Trichosporon asahii* 1188 (TA)	>500 >500	500/>500	125/125	15.62/15.62	7.81/7.81	7.81/7.81	>500/>500	0.49/1.95	31.25/31.25	7.81/7.81
	>500	>500	500	31.25	7.81	7.81	>500	1.95	125	62.5
*Aspergillus fumigatus* 231 (AF)	>500/>500	>500/>500	125/250	62.5/62.5	15.62/15.62	>500/>500	>500/>500	0.98/3.91	7.81/15.62	7.81/7.81
	>500	>500	>500	250	62.5	>500	>500	3.91	125	62.5
*Absidia corymbifera* 272 (AC)	>500/>500	>500/>500	500/500	62.5/62.5	15.62/31.25	>500/>500	>500/>500	7.81/7.81	31.25/31.25	7.81/7.81
	>500	>500	500	500	62.5	>500	>500	7.81	125	62.5
*Trichophyton mentagrophytes* 445 (TM)	>500>500	>500/>500	62.5/62.5	15.62/62.5	15.62/15.62	>500/>500	>500/>500	0.98/0.98	15.62/15.62	7.81/7.81
	>500	>500	125	125	62.5	>500	>500	1.95	15.62	62.5

Notes: ^a^ B_12_, B_14_, B_16_ mean *N*-benzyl-*N*,*N*-dimethyl-*N*-dodecylammonium bromide, *N*-benzyl-*N*,*N*-dimethyl-*N*-tetradecylammonium bromide, *N*-benzyl-*N*,*N*-dimethyl-*N*-hexadecylammonium bromide, respectively. The preparation [33] and antimicrobial efficacy of B_12–16_ has been published elsewhere. The antifungal activities against *Trichophyton mentagrophytes* 445 were determined after 72 h and 120 h of incubation.

**Table 3:** Minimum inhibitory/bactericidal concentrations of the prepared compounds (**9**–**15**) and selected derivatives of *N*-benzalkonium (B_12_–B_16_). The *in vitro* activities were determined on a panel of eight bacterial strains.

Microorganisms	MIC (μmol/L); 24 h/48 h Incubation									
	MBC (μmol/L); 48 h Incubation									
	9	10	11	12	13	14	15	B_12_^a^	B_14_^a^	B_16_^a^
*Staphylococcus aureus* CCM 451608 (SA)	125/ 125	15.62/15.62	15.62/15.62	1.95/7.81	0.98/0.98	7.81/7.81	15.62/15.62	0.49/1.95	0.98/0.98	0.98/0.98
	125	15.62	15.62	7.81	0.98	7.81	15.62	1.95	3.91	3.91
*Staphylococcus aureus* H 599608 (MRSA)	3.9/15.62	31.25/31.25	7.81/15.62	0.49/3.9	0.98/7.81	15.62/125	62.5/>250	0.49/0.49	1.95/1.95	1.95/1.95
	15.62	31.25	15.62	7.81	7.81	125	>250	0.98	3.91	3.91
*Staphylococcus epidermidis* H 696608 (SE)	62.5/125	15.62/15.62	0.98/1.95	0.98/0.98	0.98/0.98	1.95/1.95	1.95/1.95	0.49/0.49	0.98/0.98	0.49/0.49
	250	15.62	1.95	0.98	0.98	1.95	1.95	0.49	0.98	3.91
*Enterococcus sp*. J 1436508 (ES)	500/>500	62.5/125	31.25/31.25	0.98/7.81	7.81/7.81	3.9/15.62	62.5/62.5	0.49/0.98	1.95/1.95	1.95/1.95
	>500	500	62.5	7.81	7.81	15.62	62.5	0.98	7.81	3.91
*Escherichia coli* CCM 4517 (EC)	>500/>500	500/500	62.5/62.5	15.62/15.62	15.62/15.62	>500/>500	>250/>250	0.49/1.95	7.81/7.81	7.81/7.81
	>500	500	62.5	15.62	15.62	>500	>250	1.95	7.81	7.81
*Klebsiella pneumoniae* D 1175008 (KP)	>500/>500	500/500	62.5/62.5	15.62/15.62	15.62/15.62	>500/>500	>250/>250	0.49/0.49	7.81/7.81	7.81/7.81
	>500	500	62.5	15.62	15.62	>500	>250	0.49	7.81	7.81
*Klebsiella pneumoniae* J 1436808 (KP-E))	>500/>500	>500/>500	125/125	15.62/15.62	31.25/31.25	>500/>500	>250/>250	0.98/0.98	7.81/7.81	7.81/7.81
	>500	>500	125	15.62	31.25	>500	>250	0.98	7.81	7.81
*Pseudomonas aeruginosa* CCM 1961 (PA) ^c^	>500/>500	500/500	125/125	15.62/15.62	250/250	>500/>500	>250/>250	3.91/3.91	15.62/31.25	15.62/31.25
	>500	500	125	15.62	250	>500	>250	7.81	62.5	125

Notes: ^a^ B_12_, B_14_, B_16_ mean *N*-benzyl-*N*,*N*-dimethyl-*N*-dodecylammonium bromide, *N*-benzyl-*N*,*N*-dimethyl-*N*-tetradecylammonium bromide, *N*-benzyl-*N*,*N*-dimethyl-*N*-hexadecylammonium bromide, respectively. The preparation [33] and antimicrobial efficacy of B_12–16_ has been published elsewhere. The antibacterial activities against *Pseudomonas aeruginosa* CCM 1961 were determined after 72 h and 120 h of incubation.

The sensitivity of G− bacteria (the last four strains) is much lower compared to benzalkonium salts. The lower potency of compounds **11**–**13** is evident (higher MIC and MBC). However, an unexpected sensitivity of PA to compound **12** was observed. With few exceptions, compounds **9**, **10**, **14** and **15** showed no potency against G− bacteria.

### 2.3. Cytotoxicity

Cell viability assay confirmed an expected trend that increasing length of carbon chain results in higher cytotoxicity, probably due to higher lipophilicity of the drug, which facilitates penetration into the cell. Comparing the cytotoxic potential of individual analogues, none of the new compounds exceeded its benzalkonium analogue (B_12_) when it comes to the safety potential (Table 4).

**Table 4:** Cytotoxic potential of the prepared compounds (9–15) and selected derivatives of *N*-benzalkonium (B_12_–B_16_). The *in vitro* activities are expressed as IC_50_ (μmol/L) ± SEM (*n* = 3).

Cell Line	IC_50_ (μmol/L); 24 h Incubation ± SEM									
	9	10	11	12	13	14	15	B_12_^a^	B_14_^a^	B_16_^a^
CHO-K1	>1000	108 ± 5	16 ± 2	14 ± 1	7 ± 1	3.5 ± 0.1	2.9 ± 0.2	29 ± 3	24 ± 4	15 ± 1

Note: ^a^ B_12_, B_14_, B_16_ mean *N*-benzyl-*N*,*N*-dimethyl-*N*-dodecylammonium bromide, *N*-benzyl-*N*,*N*-dimethyl-*N*-tetradecylammonium bromide, *N*-benzyl-*N*,*N*-dimethyl-*N*-hexadecylammonium bromide, respectively. The cytotoxic potential of B_12–16_ has been published elsewhere [33].

## 3. Experimental Section

### 3.1. Synthesis

The pyridinium-4-aldoxime salts **10**–**14** were prepared by reaction of 4-pyridinealdoxime (**1**; 8.19 mmol) with 1-bromoalkane (**2**; 12.46 mmol) in CH_3_CN (12 mL), as shown in Scheme 1. The mixture of 4-pyridinealdoxime with 1-bromoalkane in CH_3_CN was stirred under reflux for 48 h, and the prepared salts were obtained as white crystals by crystallization from acetone, filtered, washed with acetone and allowed to dry at room temperature The second method for the synthesis (Scheme 1) of monoquaternary pyridinium-4-aldoxime salts (**3**–**9**) was as follows: Pure 4-pyridinealdoxime (**1**; 1eq 8.19 mmol) in dry ethanol (30 mL) was mixed with 1-bromoalkane (**2**; 1,4 eq 12.46 mmol). The mixture was refluxed for 48 h. The solution was evaporated under reduced pressure and the crude oily product was recrystallized from ethyl acetate, filtered, washed with ethyl acetate, and allowed to dry at room temperature.

The progress of the reaction was monitored y TLC (mobile phase ethyl acetate/methanol = 100:1). All prepared products **9**–**15** were characterized by ^1^H-NMR, ^13^C-NMR, elementary analysis and MS analysis.

Acquired yields (%), melting points (Boetius, uncorrected) and Clog*P* of prepared salts **9**–**15** are summarized in Table 1. All chemicals were reagent or higher grade of purity and were purchased from Sigma-Aldrich. The progress of the reaction was checked by Thin Layer Chromatography (TLC) (Merck Milipore Silica gel 60G/UV254, Darmstadt, Germany) with UV detection using wavelength 254 nm. The ^1^H-NMR and ^13^C-NMR spectra were recorded with a Varian Mercury-VxBB 300 with frequencies 300.07 MHz for ^1^H and 75.46 MHz for ^13^C. For ^1^H δ are given in parts per million (ppm) relative to DMSO (δ = 2.50) and for ^13^C relative to DMSO (δ = 39.43). Log*P* and Clog*P* were calculated with PC software CS ChemBioDraw Ultra 13.0 (CambridgeSoft, Cambridge, MA, USA).

*4-Hydroxyiminomethyl-1-octylpyridinium bromide* (**9**). ^1^H-NMR (300 MHz, DMSO-*d_6_*): δ 12.82 (s, 1H, OH); 9.08 (d, *J* = 6.3 Hz, 2H, 2 × ArH); 8.44 (s, 1H, CH); 8.24 (d, *J* = 6.7 Hz, 2H, 2 × ArH); 4.57 (t, *J* = 7.3 Hz, 2H, NCH_2_-); 1.97–1.81 (m, 2H, CH_2_); 1.35–1.16 (m, 10H, 5 × CH_2_); 0.85 (t, *J* = 6.5 Hz, 3H, CH_3_). ^13^C-NMR (75 MHz, DMSO-*d_6_*): δ 148.5, 145.2, 145.1, 124.2, 60.4, 49.2, 31.5, 30.7, 28.9, 25.5, 22.3, 14.1. ESI-MS: *m*/*z* 235.00 [M^+^] (counted for: [C_14_H_23_N_2_O]^+^ 235,18). Anal. Calcd. for C_14_H_23_BrN_2_O: 53.34% C; 7.35% H; 8.89% N. Found: 54.23% C; 7.31% H; 8.22% N.

*1-Decyl-4-hydroxyiminomethylpyridinium bromide* (**10**). ^1^H-NMR (300 MHz, DMSO-*d_6_*): δ 12.80 (s, 1H, OH); 9.08 (d, *J* = 6.4 Hz, 2H, 2 × ArH); 8.43 (s, 1H, CH); 8.23 (d, *J* = 6.6 Hz, 2H, 2 × ArH); 4.57 (t, *J* = 7.3 Hz, 2H, NCH_2_); 1.96–1.81 (m, 2H, CH_2_); 1.33–1.17 (m, 14H, 7 × CH_2_); 0.83 (t, *J* = 6.6 Hz, 3H, CH_3_). ^13^C-NMR (75 MHz, DMSO-*d_6_*): δ 148.5, 145.2, 145.1, 124.2, 60.4, 49.2, 31.5, 30.7, 29.1, 28.9, 25.5, 22.3, 14.1. ESI-MS: *m*/*z* 263.00 [M^+^] (counted for: [C_16_H_27_N_2_O]^+^ 263,21). Anal. Calcd. for C_16_H_27_BrN_2_O: 55.98% C; 7.93% H; 8.16% N. Found: 55.38% C; 7.62% H; 8.15% N.

*1-Dodecyl-4-hydroxyiminomethylpyridinium bromide* (**11**). ^1^H-NMR (300 MHz, DMSO-*d_6_*): δ 12.80 (s, 1H, OH); 9.09 (d, *J* = 6.4 Hz, 2H, 2 × ArH); 8.43 (s, 1H, CH); 8.23 (d, *J* = 6.7 Hz, 2H, 2 × ArH); 4.57 (t, *J* = 7.3 Hz, 2H, NCH_2_); 1.97–1.80 (m, 2H, CH_2_); 1.32–1.16 (m, 18H, 9 × CH_2_); 0.83 (t, *J* = 6.7 Hz, 3H, CH_3_). ^13^C-NMR (75 MHz, DMSO-*d_6_*): δ 148.5, 145.2, 145.1, 124.2, 60.4, 49.2, 31.5, 30.7, 29.1, 29.0, 28.9, 28.8, 28.6, 25.5, 22.3, 14.2. ESI-MS: *m*/*z* 291.00 [M^+^] (counted for: [C_18_H_31_N_2_O]^+^ 291.24). Anal. Calcd. for C_18_H_31_BrN_2_O: 58.22% C; 8.41% H; 7.54% N. Found: 57.70% C; 8.44% H; 7.54% N.

*4-Hydroxyiminomethyl-1-tetradecylpyridinium bromide* (**12**). ^1^H-NMR (300 MHz, DMSO-*d_6_*): δ 12.81 (s, 1H, OH); 9.07 (d, *J* = 6.4 Hz, 2H, 2 × ArH); 8.43 (s, 1H, CH); 8.23 (d, *J* = 6.5 Hz, 2H, 2 × ArH); 4.56 (t, *J* = 7.2 Hz, 2H, NCH_2_); 1.97–1.81 (m, 2H, CH_2_); 1.32–1.17 (m, 22H, 11 × CH_2_); 0.83 (t, *J* = 6.6 Hz, 3H, CH_3_). ^13^C-NMR (75 MHz, DMSO-*d_6_*): δ 148.5, 145.3, 145.2, 124.2, 60.2, 49.9, 31.4; 30.7, 30.1, 30.0, 29.8, 29.7, 29.3, 29.1, 28.6, 25.5, 22.3, 14.1. ESI-MS: *m*/*z* 319.00 [M^+^] (counted for: [C_20_H_35_N_2_O]^+^ 319.27). Anal. Calcd. for C_20_H_35_BrN_2_O: 60.14% C; 8.33% H; 7.01% N. Found: 59.65% C; 8.98% H; 7.02% N.

*1-Hexadecyl-4-hydroxyiminomethylpyridinium bromide* (**13**). ^1^H-NMR (300 MHz, DMSO-*d_6_*): δ 12.82 (s, 1H, OH); 9.07 (d, *J* = 6.4 Hz, 2H, 2 × ArH); 8.44 (s, 1H, CH); 8.24 (d, *J* = 6.7 Hz, 2H, 2 × ArH); 4.57 (t, *J* = 7.2 Hz, 2H, NCH_2_); 1.96–1.82 (m, 2H, CH_2_); 1.32–1.18 (m. 26H, 13 × CH_2_); 0.84 (t, *J* = 6.4 Hz, 3H, CH_3_). ^13^C-NMR (75 MHz, DMSO-*d_6_*): δ 148.5, 145.3, 145.2, 124.2, 60.2, 49.9, 31.4; 30.7, 30.1, 30.0, 29.8, 29.7, 29.6, 29.3, 29.1, 28.9, 28.6, 25.5, 22.3, 14.1. ESI-MS: *m*/*z* 347.00 [M^+^] (counted for: [C_22_H_39_N_2_O]^+^ 347.31). Anal. Calcd. for C_22_H_39_BrN_2_O: 61.81% C; 9.20% H; 6.55% N. Found: 61.52% C; 9.24% H; 6.64% N.

*4-Hydroxyiminomethyl-1-octadecylpyridinium bromide* (**14**). ^1^H-NMR (300 MHz, DMSO-*d_6_*): δ 12.82 (s, 1H, OH); 9.08 (d, *J* = 6.4 Hz, 2H, 2 × ArH); 8.44 (s, 1H, CH); 8.24 (d, *J* = 6.6 Hz, 2H, 2 × ArH); 4.57 (t, *J* = 7.3 Hz, 2H, NCH_2_); 1.97–1.81 (m, 2H, CH_2_); 1.35–1.15 (m, 30H, 15 × CH_2_); 0.84 (t, *J* = 6.7 Hz, 3H, CH_3_). ^13^C-NMR (75 MHz, DMSO-*d_6_*): δ 148.5, 145.3, 145.2, 124.2, 60.2, 49.9, 31.4; 30.7, 30.1, 30.0, 29.8, 29.7, 29.6, 29.5, 29.3, 29.2, 29.1, 28.9, 28.6, 25.5, 22.3, 14.1. ESI-MS: *m/z* 375.34 [M^+^] (counted for: [C_24_H_43_N_2_O]^+^ 375.34). Anal. Calcd. for C_24_H_43_BrN_2_O: 63.28% C; 9.51% H; 6.15% N. Found: 62.66% C; 9.67% H; 6.09% N.

*Eicosyl-4-hydroxyiminomethylpyridinium bromide* (**15**). ^1^H-NMR (300 MHz, DMSO-*d_6_*): δ 12.82 (s, 1H, OH); 9.05 (d, *J* = 6.2 Hz, 2H, 2 × ArH); 8.43 (s, 1H, CH); 8.23 (d, *J* = 6.5 Hz, 2H, 2 × ArH); 4.56 (t, *J* = 7.3 Hz, 2H, NCH_2_); 1.98–1.80 (m, 2H, CH_2_); 1.33–1.17 (m, 34H, 17 × CH_2_); 0.85 (t, *J* = 6.6 Hz, 3H, CH_3_). ^13^C-NMR (75 MHz, DMSO-*d_6_*): δ 148.5, 145.3, 145.2, 124.2, 60.2, 49.9, 31.4; 30.7, 30.1, 30.0, 29.8, 29.7, 29.6, 29.5, 29.4, 29.3, 29.2, 29.1, 28.9, 28.8, 28.6, 25.5, 22.3, 14.1. ESI-MS: *m*/*z* 403.50 [M^+^] (counted for: [C_26_H_47_N_2_O]^+^ 403.37). Anal. Calcd. for C_26_H_47_BrN_2_O: 64.58% C; 9.80% H; 5.79% N. Found: 63.94% C; 9.80% H; 5.76% N.

### 3.2. HPLC Analysis

After the preparation of the whole set of 4-PA salts differing in the length of alkyl chain, we have developed an appropriate method for their resolution in a mixture using HPLC. The HPLC system consisted of a P200 gradient pump (Spectra-Physics Analytical, Fremont, CA, USA), a 7125 injection valve—10 μL loop (Rheodyne, Cotati, CA, USA), a UV1000 detector (Spectra-Physics Analytical, Fremont, CA, USA) and CSW Chromatography Station 1.5 software (DataApex, Praha, Czech republic). For analyses a 250 × 4.6 mm I.D. Waters Spherisorb Cyano (5 µm) column was used (Supelco Inc., Bellefonte, PA, USA). The mobile phase was 45% acetonitrile and 55% water. This mixture was prepared as a 0.1 M sodium acetate solution. Finally, the pH was adjusted with acetic acid to 5000. It was delivered isocratically at a flow-rate of 1 mL/min. The absorbance was measured at 257 nm. Retention times are summarized in Table 1.

### 3.3. In-Vitro Antimicrobial Testing

#### 3.3.1. Antifungal Activity

*In vitro* antifungal activity of the prepared compounds was evaluated on a panel of eight clinical isolates of fungi, four yeasts (*C. albicans* ATCC 44859, *C. krusei* E28, *C. tropicalis* 156, *C. glabrata* 20/I) and four filamentous fungi (*Trichosporon asahii* 1188, *Aspergillus fumigatus* 231, *Absidia corymbifera* 272, *Trichophyton mentagrophytes* 445). All strains were part of the collection of fungal strains and are deposited at the Department of Biological and Medical Sciences, Faculty of Pharmacy, Charles University, Hradec Kralove, Czech Republic. The ATCC strains *C. albicans* ATCC 90028, *C. parapsilosis* ATCC 22019, and *C. krusei* ATCC 6258 served as the quality control strains.

All the isolates were maintained on Sabouraud dextrose agar prior to being tested. Minimum inhibitory concentration (MIC) was determined by the modified microdilution format of the CLSI M27-A3 and M38-A2 for yeasts and filamentous fungi, respectively [42,43]. Dimethyl sulfoxide (Sigma, Prague, Czech Republic) served as a diluent for all compounds and its final concentration did not exceed 2%. RPMI 1640 (KlinLab, Prague, Czech Republic) medium supplemented with l-glutamine and buffered with 0.165 M morpholinepropanesulfonic acid (Sigma-Aldrich, Prague) to pH 7.0 by 10 M NaOH was used as a test medium. The wells of the microdilution tray contained 200 μL of the RPMI 1640 medium with two fold serial dilutions of the prepared compounds (500–0.49 μmol/L) and were inoculated with 10 μL of suspension. The fungal inoculum in RPMI 1640 was prepared to give a final concentration of 5 × 10^3^ ± 0.2 cfu/mL and 5 × 10^4^ ± 0.5 cfu/mL for yeasts and moulds, respectively. The trays were incubated at 36 °C ± 1 °C and MIC was read visually and spectrophotometrically (OD 450 nm) for filamentous fungi and yeasts respectively after 24 and 48 h. The MIC values for the dermatophytic strain (*T. mentagrophytes*) were determined after 72 h and 120 h. The MICs were defined as 80% inhibition (IC_80_) of the growth of control. Minimum fungicidal concentration (MFC) was established for all compounds tested as the concentration which provided a decrease in the number of colonies by ≥99.9% after subculturing of a 100 μL aliquot of each well with maximum growth of 20% of control.

#### 3.3.2. Antibacterial Activity

The *in vitro* antibacterial activity of the prepared compounds was tested on a panel of eight bacterial strains (*Staphylococcus aureus* CCM 4516/08, *S. aureus* MRSA H 5996/08, *S. epidermidis* HK6966/08, *Enterococcus* sp. HK14365/08, *Escherichia coli* CCM 4517, *Klebsiella pneumoniae* D 11750/08, *K. pneumoniae* J 14368/08, and *Pseudomonas aeruginosa* CCM 1961). The ATCC strains also served as the quality control strains; the rest of them were clinical isolates from the patients and are deposited at the Department of Biological and Medical Sciences, Faculty of Pharmacy, Charles University, Hradec Kralove, Czech Republic. Before testing the strains were passaged on Mueller-Hinton Agar (HiMedia, Cadersky-Envitek, Prague, Czech Republic).

Minimum inhibitory concentration (MIC) of the prepared compounds was determined by the microdilution broth method modified according to standard M07-A07 [44]. Mueller-Hinton Broth (MH, HiMedia, Cadersky-Envitek, Prague, Czech Republic) adjusted to pH 7.4 (±0.2) was used as the test medium. DMSO served as a diluent for all compounds and its final concentration did not exceed 2% in the test medium. The wells of the microdilution tray contained 200 μL of the MH broth with twofold serial dilutions of the compounds (500–0.49 μmol/L) and were inoculated with 10 μL of bacterial suspension. A bacterial inoculum in sterile water was prepared to match 0.5 McFarland scale (1.5 × 10^8^ CFU/mL). The MIC values were read visually after 24 h and 48 h incubation at 36 °C ± 1 °C; for *Pseudomonas aeruginosa* CCM 1961, the MIC was determined after 72 h and 120 h of incubation. The MIC was defined as complete inhibition of growth. Minimum bactericidal concentration (MBC) was established for all compounds tested as the concentration that provided a decrease in the number of colonies by ≥99.9% after subculturing of a 100 μL aliquot of each well without visible growth.

### 3.4. Cytotoxicity

Standard MTT assay (Sigma Aldrich, Prague, Czech Republic) was used according to the manufacturer’s protocol on the CHO-K1 cell-line (Chinese hamster ovary, ECACC, Salisbury, UK) in order to compare the cytotoxic effect of the studied compounds. The cells were cultured according to ECACC recommended conditions and seeded at a density of 8000 per well. Briefly, the tested compounds were dissolved in DMSO and subsequently in the growth medium (F-12) supplemented with 10% FBS and 1% penicillin/streptomycin so that the final concentration of DMSO did not exceed 0.5% (v/v). Cells were exposed to the tested compounds for 24 h. The medium was then replaced by a medium containing 10 μM of MTT and the cells were allowed to produce formazan for another 3 h under surveillance. Thereafter, the medium with MTT was removed and crystals of formazan were dissolved in DMSO (100 μL). Cell viability was assessed spectrophotometrically by the amount of formazan produced. Absorbance was measured at 570 nm with 650 nm reference wavelength on Synergy HT (BioTek, Winooski, VT, USA). IC_50_ was then calculated from the control-subtracted triplicates using non-linear regression (four parameters) of GraphPad Prism 5 software. The final IC_50_ and SEM value was obtained as the mean of three independent measurements.

## 4. Conclusions

The whole set of pyridine-4-aldoxime based quaternary ammonium salts with differing length of alkyl side chain was synthesized (**9**–**15**). We have described two possible conditions for the preparation. Both methods are usable for the preparation and no significant difference in the yields was observed. The compounds were analyzed by NMR, EA and MS analysis and the log*P* was calculated for each compound. Furthermore, an HPLC experimental protocol was found that was fully applicable for purity evaluation and could be easily used for characterization of mixtures of all homologues. Antimicrobial (antifungal and antibacterial) activity evaluation confirmed that compounds having an alkyl chain of C_12_–C_16_ outperformed the rest of the tested compounds. Yeast-type fungi were the most sensitive towards those analogues whereas the C_16_ analogue was completely ineffective against filamentous fungi. Antibacterial assessment revealed versatility of C_14_ and relatively high efficacy of C_16_ against G+ strains and C_14_ against G− strains. Notably, none of the studied compounds exceeded the efficacy and versatility of the benzalkonium C_12_ analogue, and benzalkonium analogues also showed a lower effect in the cell viability assay.
